# Prediction of Recurrence of Completely Resected Pancreatic Solid Pseudopapillary Neoplasms in Pediatric Patients: A Single Center Analysis

**DOI:** 10.3390/children8080632

**Published:** 2021-07-25

**Authors:** Joonhyuk Son, Wontae Kim, Jeong-Meen Seo, Sanghoon Lee

**Affiliations:** 1 Department of Pediatric Surgery, Hanyang University College of Medicine, Seoul 04763, Korea; cross0911@gmail.com; 2 Department of Surgery, Samsung Medical Center, Sungkyunkwan University School of Medicine, Seoul 06354, Korea; wnt.kim@samsung.com (W.K.); jm0815.seo@samsung.com (J.-M.S.)

**Keywords:** solid pseudopapillary neoplasm, pancreas, pediatric, recurrence, lymph node metastasis

## Abstract

Background: Many previous studies have investigated the risk factors for the recurrence of pancreatic solid pseudopapillary neoplasms (SPNs), although a consensus has not yet been reached, despite this effort. We aimed to identify the predictive factors for recurrence in patients with SPNs who underwent complete surgical resection of the tumor. Methods: We retrospectively analyzed the records of pediatric patients with SPNs who underwent surgical resection at a single center between 2001 and 2018. Results: During the study period, 47 patients with SPNs underwent radical resection of the tumor. The median age of the patients was 14 (8–18) years. R0 resection was confirmed in every case and none of the patients presented with systemic metastasis at the time of diagnosis. The median follow-up period was 53.1 (30.8–150.8) months. Of the 47 patients, only two (4.2%) experienced recurrence. Using comparative analysis, we found that some factors such as a large tumor size, peripancreatic tissue invasion, and capsule invasion did not increase the risk of recurrence of SPNs. Lymph node metastasis was the only significant factor for recurrence in our study (*p* = 0.043). Conclusion: During our single center analysis, we found that only lymph node metastasis was a predictive factor for recurrence of SPNs among patients who underwent complete tumor resection. Long-term follow-up is required to determine whether SPNs will recur if lymph node metastasis is observed after surgery. Furthermore, therapeutic benefits of routine lymphadenectomy or sentinel lymph node biopsy should be investigated in future studies to reduce the risk of recurrence in patients with SPNs.

## 1. Introduction

Solid pseudopapillary neoplasms (SPNs) of the pancreas are rare tumors, which usually occur in young women [1,2]. Most patients with SPNs undergo surgical resection and have excellent outcomes with a good prognosis [3]. However, postoperative recurrences with or without distant metastasis has been reported in most of the literature, ranging from 1% to 9% [4,5,6,7,8,9,10,11,12]. Despite the good survival outcomes, recurrence and metastasis still can be a disease burden for the patient, especially in pediatric patients, who have a longer life expectancy than adults. Numerous studies have proposed possible clinicopathological factors associated with the recurrence of SPNs [9,13,14,15,16,17,18]. However, it is difficult to predict the malignant potential and risk of recurrence. Thus, no consensus has been reached regarding surgical strategies to prevent recurrence.

Considering these circumstances, we aimed to identify predictive factors that are associated with the recurrence of SPNs and to determine the appropriate strategy to reduce the risk of recurrence after surgery.

## 2. Materials and Methods

All patients younger than 18 years who were diagnosed with SPNs and underwent surgical resection to treat them at Samsung Medical Center between 2001 and December 2018 were included in this study. Patient data such as demographic data, clinical characteristics, pathological information, and postoperative clinical outcomes were retrospectively collected after reviewing the patients’ medical records.

All of the patients underwent complete surgical resection of the tumors. None of the patients presented with synchronous metastasis at the time of initial diagnosis and surgery. Lymphadenectomy was not routinely performed at our center; however, when suspicious lymphadenopathy was identified during surgery or when tumor infiltration around the adjacent tissues occurred, some of the lymph nodes around the tumor were removed. After discharge, patients were followed up at the outpatient clinic with computed tomography (CT) scan or ultrasonography every 6 months or a year.

The Student’s t-test was performed to analyze the continuous variables, and the chi-squared test was used to analyze categorical variables. Statistical significance was set at *p* < 0.05.

This study was approved by the Institutional Review Board at Samsung Medical Center (IRB File No. 2021-06-080).

## 3. Results

During the study period, 47 patients were diagnosed with SPNs, and surgical resection was performed on each patient. Their clinicopathological characteristics are summarized in Table 1. The median age of the patients was 14 (range, 8–18) years. Among the patients, 5 were males and the remaining 42 were females. Seventeen patients underwent pylorus-preserving pancreaticoduodenectomy (PPPD) for tumors in the head of pancreas, and 29 underwent distal pancreatectomy with or without splenectomy. Two patients had suspicious tumor invasion to the adjacent small intestine, and combined resection of the affected portion of the intestine was performed during the surgery. None of the cases experienced mortality or serious complications requiring surgical intervention or readmission. The median follow-up period was 53.1 (30.8–150.8) months.

### 3.1. Histopathological Characteristics

Thirty patients (63.8%) had tumors located in the body or tail of the pancreas. The mean tumor size was 6.3 (1.4–13) cm. Each patient had their tumors curatively resected, and negative resection margins (R0 resection) were confirmed for every cases. No angio-vascular invasion or perineural invasion was observed in our cases. One patient had lymph node metastases. The most common microscopic malignant feature was peripancreatic tissue invasion, which was reported in 31.9% of patients.

### 3.2. Recurrence

Of the 47 patients, two experienced recurrence (4.2%). The analysis of clinical factors for the recurrence of SPNs is shown in Table 2. According to the comparative analysis, large tumor size, peripancreatic tissue invasion, and capsule invasion did not increase the risk of recurrence of SPNs, although lymph node metastasis was a significant risk factor for recurrence (*p* = 0.043) (Table 2). We reviewed the clinical course of two patients who experienced recurrence after surgery. Patient A had lymph node metastases (two metastasized lymph nodes out of three retrieved lymph nodes) at the time of the initial operation. A follow-up CT scan performed 47 months after the surgery showed liver metastasis, which was treated with partial liver resection. Patient A was disease-free at the last follow-up. The other patient (Patient B) had no retrieved lymph nodes at the time of initial surgery. There were no suspicious microscopic malignant features in patient B. However, 38 months after the initial surgery, a follow-up CT scan showed suspicious regional lymph node metastases around the remnant pancreas and the patient underwent excision of the recurrent lymph node metastases. Despite the second operation, the patient developed peritoneal metastasis during the follow-up period.

To confirm the association between lymph node status and systemic recurrence, comparative analysis was performed among patients who have had lymphadenectomy done (Table 3). Forty patients underwent lymph node surgery, and their nodal status was reviewed by the pathologists. Tumor size and pathologic malignant features had no association with lymph node metastasis. Systemic recurrence was significantly observed in patients with lymph node metastasis (*p* = 0.001).

## 4. Discussion

SPNs of the pancreas are rare tumors with good prognosis and excellent overall survival. However, most studies report a certain rate of tumor recurrence either with or without distant metastasis after surgical resection. According to the WHO criteria, pathological factors such as perineural invasion, angioinvasion, and deep invasion into the surrounding tissues may be indicators of malignant behavior in SPNs [9,18,19]. In addition, numerous studies have assessed risk factors for the recurrence of SPNs after surgery [9,13,14,15,16,17,18]. However, these factors do not always correlate with recurrence, and patients with tumors without ‘malignant’ factors may also experience recurrence. In 2010, the WHO classified all SPNs as low-grade malignant neoplasms regardless of microscopic malignant features [9,18].

Most of the factors associated with recurrence thus far are pathological, which means that it is difficult to predict the malignant behavior of SPNs preoperatively. In 2014, Kang et al. conducted a large multicenter study using nationwide data [9]. They reported that tumors larger than 8 cm, tumors with microscopic malignant features, and stage IV disease were significant risk factors for the recurrence of SPNs. In 2018, Gao et al. conducted a meta-analysis assessing the risk factors for the recurrence of SPNs [18]. They concluded that patients with tumors larger than 5cm, lymphovascular invasion, lymph node metastasis, synchronous metastasis and positive margins were more likely to experience recurrence of SPNs. Besides the size of the tumor and the tumor stage (synchronous metastasis), all these factors are observed postoperatively, making it difficult for surgeons to predict malignancy and decide the extent of the surgical resection of SPNs during surgery. Tang et al. suggested that peripancreatic lymphadenopathy on preoperative radiologic images was associated with malignancy in patients with SPN [20]. Therefore, the author proposed that lymphadenectomy should be performed in patients with suspicious lymphadenopathy on preoperative imaging. According to our results, patient A did not have suspicious lymphadenopathy on preoperative imaging. This means that lymph node metastasis can be present without being observed on preoperative images.

The optimal surgical strategy to treat SPNs is controversial. Complete radical resection of the tumor seems to be the treatment of choice for SPNs. However, due to the indolent course of SPNs, several studies have proposed less aggressive treatment. Minimal surgical resection, such as enucleation and local excision of the tumor, can reduce postoperative morbidity and prevent postoperative endocrine and exocrine insufficiency [19,21,22,23,24]. In our center, all patients were treated with complete radical resection of the tumor with curative intent. Tumors with suspicious adjacent organ invasions were treated with synchronous resection of adjacent organs at the time of surgery. However, although all our surgical cases achieved R0 status, recurrence still occurred. We should note that both our recurrent patients had lymph node metastases before the tumor metastasized to distant organs, and there were no other suspicious histopathological factors that indicated malignancy at the initial surgery. Considering the pattern of distant metastasis in our cases, we can assume that SPNs metastasize through the lymphatic systems and lymph nodes, and aggressive lymphadenectomy might reduce the risk of recurrence. However, there is insufficient evidence to generalize this theory based on these two cases. To assess the clinical value of lymph node status in the treatment of SPNs, future prospective studies regarding the effect of lymphadenectomy or sentinel lymph node biopsy might be required.

The decision to perform aggressive surgeries in patients with metastatic disease is another surgical enigma for many surgeons. Few studies have reported patients surviving with metastatic disease for an extended period with non-surgical treatment (chemotherapy or radiotherapy) or even without surgery [25,26,27]. However, Jutric et al. reported that patients who underwent surgical resection to treat distant metastatic tumors had better survival outcome than those who did not undergo surgery [28]. In addition, many other studies have proposed that metastatic SPNs should be treated with aggressive surgeries to achieve improved long-term survival rates [6,9,29,30]. For pediatric patients with a longer life expectancy than adults, it is important to use every surgical strategy available to achieve longer survival in patients with metastatic and recurrent SPNs. However, due to the possibility of postoperative morbidity and long-term complications, aggressive surgical treatment might be another disease burden for young patients. Thus, to develop the best treatment strategy for young patients with recurrence or distant metastasis, their quality of life should be assessed, along with their survival outcomes.

This study has several limitations. First, as we explained earlier, it is difficult to generalize our results due to the small number of cases in our center. Second, neither angioinvasion nor perineural invasion were observed in our cases. If these pathologic factors had been identified, the outcomes might have been different. Third, this was a retrospective single-center review, and the results do not differ from those of previous reports. However, while most previous studies included cases with R1 status, patient who underwent limited surgeries such as enucleations, and synchronous metastatic cases, our series only included cases that underwent radical resection of the SPNs, all of whom achieved R0 status pathologically. This means that our results might help surgeons to predict the recurrence of SPNs among patients who had their tumors completely resected with R0 resection.

In conclusion, our analysis showed that lymph node metastasis was the only pathological factor associated with the recurrence of SPNs among patients who underwent complete tumor resection. When lymph node metastasis is identified at initial surgery, careful follow-up is needed, and the patient must be made aware that a possibility of recurrence exists. Furthermore, the therapeutic benefits of lymph node biopsy or routine lymphadenectomy to reduce the risk of the recurrence of SPNs should be discussed in future prospective studies.

## Figures and Tables

**Table 1:** Demographic characteristic of the patients.

	Overall (*n* = 47)
Age at operation (years, median)	14 (8–18)
Sex (%)	
Male	5 (10.6)
Female	42 (89.4)
Tumor size (cm, mean)	6.3 (1.4–13)
Tumor location (%)	
Head	17 (36.2)
Body and Tail	30 (63.8)
Type of operation (%)	
PPPD	17 (36.2)
Distal Pancreatectomy	29 (61.7)
Central Pancreatectomy	1 (2.1)
Macroscopic features (%)	
Adjacent organ infiltration	2 (4.3)
Microscopic features (%)	
Benign	31 (66.0)
Malignant	16 (34.0)
Microscopic malignant features (%)	
Peripancreatic soft tissue involvement	15 (31.9)
Capsule invasion	1 (2.1)
Lymph node metastasis	1 (2.1)
Perineural invasion	0 (0)
Angiovascular invasion	0 (0)
Distant metastasis	0 (0)
R1 resection (Positive margin)	0 (0)
Follow up duration (months, median)	53.1 (30.8–150.8)

PPPD, pylorus preserving pancreaticoduodenectomy.

**Table 2:** Clinical factors associated with recurrence.

	No Recurrence (*n* = 45)	Recurrence (*n* = 2)	*p*-Value
Age at operation (years, median)	13.6 ± 2.1	13.0 ± 4.2	0.701
Sex (%)			1
Male	5 (11.1)	0 (0)	
Female	40 (88.9)	2 (100)	
Tumor size (%)			1
≤5 cm	14 (31.1)	0 (0)	
>5 cm	31 (68.9)	2 (100)	
Tumor size (%)			0.481
≤8 cm	33 (73.3)	1 (50)	
>8 cm	12 (26.7)	1 (50)	
Tumor location (%)			0.528
Head	17 (37.8)	0 (0)	
Body and Tail	28 (62.2)	2 (100)	
Macroscopic features (%)			
Adjacent organ infiltration	2 (4.4)	0 (0)	1
Microscopic malignant features (%)	15 (33.3)	1 (50)	1
Peripancreatic soft tissue involvement	14 (31.1)	1 (50)	0.541
Capsule invasion	1 (2.2)	0 (0)	1
Lymph node metastasis	0 (0)	1 (50)	0.043
Other microscopic features (%)			
Necrosis	32 (71.1)	1 (50)	0.512
Hemorrhage	34 (75.6)	1 (50)	0.45
Calcification	6 (13.3)	0 (0)	1

**Table 3:** Association between clinicopathological factors and positive lymph nodes.

	Negative Lymph Node (*n* = 38)	Positive Lymph Node (*n* = 2)	*p*-Value
Tumor size (cm, mean)	7.21 ± 2.87	8.25 ± 2.47	0.623
Tumor size (%)			1
≤5cm	10 (26.3)	0 (0)	
>5cm	28 (73.7)	2 (100)	
Tumor size (%)			1
≤8cm	26 (68.4)	1 (50)	
>8cm	12 (31.6)	1 (50)	
Macroscopic features (%)			
Adjacent organ infiltration	2 (5.3)	0 (0)	1
Microscopic malignant features (%)	14 (36.8)	1 (50)	1
Peripancreatic soft tissue involvement	13 (34.2)	1 (50)	1
Capsule invasion	1 (2.6)	0 (0)	1
Systemic recurrence (%)	0 (0)	2 (100)	0.001

## Data Availability

The data presented in this study are available on request from the corresponding author.
